# Risk scores to predict decreased glomerular filtration rate at 10 years in an Asian general population

**DOI:** 10.1186/s12882-017-0653-z

**Published:** 2017-07-17

**Authors:** Krittika Saranburut, Prin Vathesatogkit, Nisakron Thongmung, Anchalee Chittamma, Somlak Vanavanan, Tuangrat Tangstheanphan, Piyamitr Sritara, Chagriya Kitiyakara

**Affiliations:** 1 Cardiovascular and Metabolic Center, Faculty of Medicine, Ramathibodi Hospital, Mahidol University, Bangkok, 10400 Thailand; 2 Department of Medicine, Faculty of Medicine, Ramathibodi Hospital, Mahidol University, 270 Rama 6 Rd, Bangkok, 10400 Thailand; 3 Research Center, Biochemistry and Chemical Analysis Unit, Faculty of Medicine, Ramathibodi Hospital, Mahidol University, Bangkok, 10400 Thailand; 4 Division of Clinical Chemistry, Department of Pathology, Faculty of Medicine, Ramathibodi Hospital, Mahidol University, Bangkok, 10400 Thailand; 5 Medical and Health Office, Electricity Generating Authority of Thailand, Bangkruay, Nonthaburi, 11130 Thailand

**Keywords:** Asian, Cohort, Chronic kidney disease, EGAT, Population, Risk score, Thai

## Abstract

**Background:**

Asians have among the highest prevalence of chronic kidney disease (CKD) or end-stage renal disease in the world. A risk score capable of identifying high risk individuals at the primary care level could allow targeted therapy to prevent future development of CKD. Risk scores for new CKD have been developed in US general populations, but the impact of various risks factors for development of CKD may differ in Asian subjects. In this study, we aimed to develop risk models and simplified risk scores to predict the development of decreased glomerular filtration rate (GFR) at 10 years in an Asian general population using readily obtainable clinical and laboratory parameters.

**Methods:**

Employees of EGAT (The Electric Generating Authority of Thailand) were studied prospectively. Multivariable logistic regression models were used to assess risk factors and used to derive risk models and risk scores for developing decreased GFR at 10 years: Model 1 (Clinical only), Model 2 (Clinical + Limited laboratory tests), and Model 3 (Clinical + Full laboratory tests). The performance of the risk models or risk scores to predict incident cases with decreased GFR were evaluated by tests of calibration and discrimination.

**Results:**

Of 3186 subjects with preserved GFR (eGFR ≥60) at baseline, 271 (8.5%) developed decreased GFR (eGFR < 60) at 10 years. Model 1 (Age, sex, systolic blood pressure, history of diabetes, and waist circumference) had good performance (χ^2^ = 9.02; AUC = 0.72). Model 2 (Age, Sex, systolic blood pressure, diabetes, glomerular filtration rate) had better discrimination (χ^2^ = 10.87, AUC = 0.79) than Model 1. Model 3 (Model 2+ Uric acid, Hemoglobin) did not provide significant improvement over Model 2. Based on these findings, simplified categorical risk scores were developed for Models 1 and 2.

**Conclusions:**

Clinical or combined clinical and laboratory risk models or risk scores using tests readily available in a resource-limited setting had good accuracy and discrimination power to estimate the 10-year probability of developing decreased GFR in a Thai general population. The benefits of the risk scores in identifying high risk individuals in the Thai or other Asian communities for special intervention requires further studies.

**Electronic supplementary material:**

The online version of this article (doi:10.1186/s12882-017-0653-z) contains supplementary material, which is available to authorized users.

## Background

Chronic kidney disease (CKD) and decreased glomerular filtration rate (GFR) are both associated with elevated risks for end-stage renal disease (ESRD), cardiovascular disease, and death [1, 2]. The incidence of CKD has increased worldwide with important public health and economic implications especially in developing countries where resources are limited. Despite the high prevalence rate, CKD awareness rates are often very low in the community as early CKD is usually asymptomatic [3, 4]. As a consequence, CKD is not frequently detected until it has already advanced, and opportunities for intervention are lost. A risk score that identifies those at higher risk for future CKD has been proposed as a stratification and prediction device [5]. Cardiovascular risk scores, such as the Framingham score, [6] have influenced public health policy in the primary prevention of cardiovascular diseases and have been tested in many populations [7]. A simple and accurate renal risk score would lead to targeted medical management at the primary care level to individuals at the highest risk for future CKD.

Asians represent 60% of the global population and have among the highest prevalence of CKD in the world [8]. Asian countries include developed and low to middle-income countries with different risk factors for CKD development [9, 10]. In low to middle-income countries, the burden of disease is changing from infections towards chronic lifestyle-related diseases as a result of demographic transition and urbanization. In 2011, Thailand was reclassified by the World Bank from a lower-middle income to a higher-middle-income country. Over the last decade, the numbers of patients with ESRD have increased by an order of magnitude. Data from the Thailand Renal Replacement Therapy registry reported that the numbers of patients on renal replacement increased from 30 per million people in 1997 to 1199 per million people in 2014 (file:///E:/TRT%202007–2017/1.TRT-report−2014-_3–11-59_-final_pdf). This staggering increase is both due to public health policy changes as well as due to higher rates of CKD.

Risk scores for incident CKD have been developed in the US general populations [11, 12], but there are limited information on risk models that allow long term predictions of incident CKD in Asians. Since risks factors for CKD and decreased GFR in populations from Asian countries including Thailand may differ from Caucasian populations, risk scores developed in an Asian community may be more appropriate to evaluate risks in Asian populations. In this study, we aimed to evaluate risk predictors and develop risk models and risk scores for developing decreased GFR at 10 years follow-up in a Thai general population cohort. We hypothesized that new cases of decreased GFR may be predicted by a risk score containing a subset of clinical variables. Because subjects from low to middle-income Asian countries often have limited resource and less access to routine medical checkups than their Western counterparts, we have developed a risk score based on clinical and simple laboratory parameters easily assessed in the primary care setting and compared them to a score based on more expanded, but still standard laboratory work up.

## Methods

### Study participants

The subjects were employees of EGAT (The Electric Generating Authority of Thailand) who volunteered to participate in a health survey. All participants completed a medical evaluation and had routine laboratory investigations including urinalysis. Blood was drawn after a 12 h fast. The details of the EGAT study cohorts and the study protocols have previously been described [13]. In summary, there were 3 EGAT cohorts in total. In 1985, 3499 workers of EGAT (half of the total employees) were randomly enrolled as EGAT 1 cohort. In 1998, 2999 employees were randomly enrolled as EGAT 2 cohort. The age range of both EGAT 1 and EGAT 2 was selected as 35–54 years old. In 2009, 2584 participants were recruited to the EGAT 3 cohort, but the age range was expanded to 25–54 years old. Participants in different EGAT cohorts did not overlap. The response rates in the first and second cohorts were >95%, but in EGAT 3 it dropped to 76%. The major reasons for this reduced response rate are thought to be that some selected participants were due to leave for periodic work in the sites outside Bangkok, and the fear of contracting swine flu in examination centers in 2009. The mean ages were not different between responders and non-responders (each was 41 years; *P* = 0.78), but responders were less likely to be male (74 vs 78%; *P* = 0.004). The EGAT 1 cohort was resurveyed in 1997, 2002, 2007, and 2012. The EGAT 2 cohort was resurveyed in 2003, 2008, and 2013. EGAT 3 was resurveyed in 2014. Each time, the same individuals were contacted by telephone and invitation letter to attend the follow-up examination, or else information about the cause of death was sought for those known to have died during the interim period. At each follow-up visit, the subjects underwent similar medical evaluations and had routine laboratory investigations as the baseline visit. The EGAT studies were conducted in accordance with the Helsinki Declarations and approved by the Ethics Committee, Ramathibodi Hospital, Mahidol University, Thailand. Written informed consent was obtained.

#### Derivation cohort

**Fig. 1:**
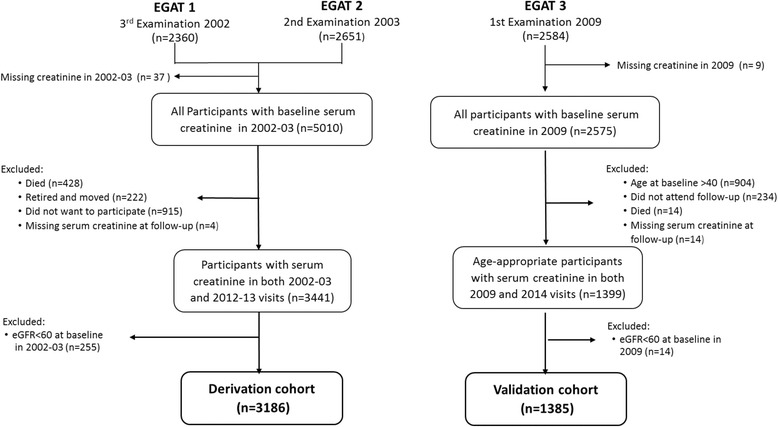
Flow chart of the Derivation and the Validation cohorts

The Derivation cohort included subjects from EGAT 1 and EGAT 2 cohorts. The 2002–2003 period (EGAT 1 3rd examination and EGAT 2 2nd examination) was selected to be the baseline of the study to ensure a broad starting age range of approximately 40–70 years old. The outcome was evaluated 10 years later in 2012–13 (EGAT 1 5th examination and EGAT 2 4th examination). Of EGAT 1 (*n* = 2360) and EGAT 2 (*n* = 2651) participants who attended the baseline 2002–2003 examination, subjects (*n* = 37) with missing baseline creatinine data were excluded leaving a total of 5010 subjects with serum creatinine at baseline in 2002–2003. The Derivation cohort dataset was derived from participants with preserved GFR (estimate glomerular filtration rate (eGFR) ≥ 60 mL/min/1.73m^2^) at baseline who attended both the examinations in 2002–2003 and 2012–13 (EGAT 1 5rd examination and EGAT 2 4nd examination) (Fig. 1).

#### Validation cohort

The Validation cohort dataset was derived from EGAT 3 cohort participants with preserved GFR (eGFR ≥ 60) at baseline in 2009 (EGAT 3 1st examination) who were followed up 5 years later in 2014 (EGAT 3 2nd examination). Participants younger than 40 years old at baseline were excluded to maintain similar cut-off age as the Derivation cohort (Fig. 1).

### Outcome definition

Incident cases with decreased eGFR *(Decreased GFR)* refers to subjects with preserved GFR (eGFR ≥60) at baseline who subsequently developed decreased GFR (eGFR < 60 mL/min/1.73m^2^) at the 10 year follow-up. The outcome is a modification from the KDIGO definition of CKD stage 3–5. The difference being that the kidney function was measured once and the presence of decreased eGFR was not confirmed to be present for greater than 3 months [14].

### Covariate assessment

An average of two blood pressure measurements in seated position were used. Hypertension was defined as systolic blood pressure (SBP) ≥ 140 mmHg or diastolic blood pressure (DBP) ≥ 90 mmHg or use of oral antihypertensive medication [15]. A positive history of diabetes was ascertained from subject testimony or the use of medications. Diabetes mellitus (DM) was defined as a fasting glucose of ≥126 mg/dl or a positive history of diabetes [16] Smokers were defined according to current status within the last 12 months. History of cardiovascular disease included subjects with previous peripheral vascular disease, heart muscle, heart attack and stroke. Waist circumference was measured midway between the lowest ribs and the iliac crest. Body mass index was defined as weight in kilograms divided by the square of height in meters. There were less than 5% missing data for the covariates and these were regarded as missing.

### Laboratory measurements and GFR estimation

Cholesterol, triglycerides, and HDL cholesterol were determined using enzymatic methods as published [17] LDL cholesterol was calculated using the Friedewald equation [18]. Serum creatinine (sCr) was measured by the enzymatic assay on the Vitros 350 analyzer (Ortho-Clinical Diagnostics, USA) using IDMS-Standard Reference Material (SRM) 967 as the standard [19]. The coefficient of variations for low, high sCr were 1.64%, 0.41%. Urine protein was detected by urinalysis reagent strip (Bayer, Indiana, USA). Estimate glomerular filtration rate (eGFR) was calculated according to two-level race variable Chronic Kidney Disease–Epidemiology Collaboration (CKD-EPI) equation [20] using the non-black coefficient as this is now the preferred equation in the general population [2].

## Statistical analysis

Continuous data is reported as mean (± SD). Categorical data is presented as percentages. Continuous variables were compared using Student’s t test. Categorical variable were compared using Chi-square test. The baseline data of the Derivation cohort were used to predict risk for *Decreased GFR* defined by CKD-EPI after excluding patients with eGFR <60 at baseline.

### Risk models

Multivariable logistic regression models were used to assess risk factors for *Decreased GFR*, selected from earlier reports [11, 21] Variables were sequentially added in a pre-specified order and incorporated using a *P* < .05 threshold for entry and retention in the final model. Three analysis models were used:

- *Model 1* contained only readily available clinical variables previously linked to CKD without the use of laboratory parameters. In order of entry, the candidates were: age, sex, history of diabetes, systolic blood pressure, waist circumference, current smoking (yes/no), and history of cardiovascular disease
- *Model 2* comprised of Model 1 plus baseline eGFR and blood sugar as linear parameters.
- *Model 3* comprised Model 2 and other biochemical parameters including uric acid, hemoglobin and HDL.

Because urine screening is not routinely performed in Thailand, we evaluated alternative models to assess the benefit of urinalysis in which dipstick proteinuria (defined as 1+ or higher) was also calculated in addition to existing models. Additional analyses were also performed in which hypertension was substituted for SBP and BMI was substituted for waist circumference.

### Risk scores

Variables were dichotomized to enhance clinical utility. A risk scoring system for CKD was developed using previously established methods. In brief, each variable was assigned points proportional to the product of its regression coefficient from the multiple logistic regression model for CKD (described above) and the measured value of that variable [6, 22].

The performance of the risk models or risk scores to predict *Decreased GFR* were evaluated by tests of calibration and discrimination. *Calibration* measures how closely predicted outcomes agree with actual outcomes. The risk models were used to divide subjects into deciles of predicted risk for *Decreased GFR*. Differences of predicted and observed rate were compared using Hosmer-Lemeshow chi-square test [23]. Chi-square values <20 generally support adequate fit. *Discrimination* is the ability of a prediction model to separate those who develop *Decreased GFR* from those who do not. We quantified this by calculating the *c* statistic, analogous to the area under a receiver operating characteristic curve (AUC) [24]. This value represents an estimate of the probability that a model assigns a higher risk to those who develop *Decreased GFR* than to those who do not. AUC varies from 0.5 (no discrimination) to 1.0 (perfect discrimination) and is reported as AUC (95% confidence interval). AUC of different models were compared using Delong test [25].

### Sensitivity analyses

As there is a day to day variation in eGFR, persons with borderline eGFR may at reevaluation have eGFR slightly lower than 60 just due to random variation. We tested the performance of the risk models after excluding *Decreased GFR* cases with an eGFR decline of less than 5 ml/min over 10 years as a sensitivity analysis.

We also evaluated the performance of the risk models using the four-level race variable CKD-EPI equation with Asians coefficients (×1.052) to calculate eGFR as this equation has been proposed by some investigators as a valid equation in Asian subjects [26].

### Internal and External validation

Internal validation of the c-statistics was performed using bootstrapping with 1000 replications of individuals sampled with replacement. The Somer’D correlation was used to estimate the correlation between the observed and predicted values for *Decreased GFR*, called D_boot_ [27]. *Calibration bias* of the model was assessed by subtracting the original Somer’D correlation from the mean D_boot_.

External validation of the performance of the Risk Score developed in the Derivation Cohort from EGAT 1 and EGAT 2 participants for predicting *Decreased GFR* was evaluated in the Validation Cohort composed of EGAT 3 participants.

Analyses were performed with SPSS version 11.5 (SPSS Inc.; Chicago, IL). Comparisons of AUCs were performed with Stata 14 (Stata Corp LP, College Station, TX).

## Results

### Baseline characteristics of the Derivation cohort and the incidence of *Decreased GFR*

Of EGAT 1 and EGAT 2 participants with serum creatinine (*n* = 5010) at baseline in 2002–2003, 428 subjects died, 222 retired and moved, 915 did not want to participate and four subjects had missing serum creatinine leaving 3441 participants with complete data for both 2002–2003 and 2012–2013 visits (Fig. 1). These subjects with complete follow-up data were 1 year younger, had slightly lower proportions of males, diabetes, hypertension, proteinuria compared to the total participants with serum creatinine in 2002–2003. The mean eGFR differed by 0.9 ml/min/1.73 m^2.^ Although these differences were statistically significant, the actual differences were generally not large (Additional file 1: Table S1).

Of the 3441 subjects with complete data for both visits, subjects with eGFR < 60 at baseline (*n* = 255) were excluded leaving 3186 patients for analysis as the Derivation cohort. Baseline characteristics of the Derivation cohort are shown in Table 1. All subjects are Thais or Thai-Chinese. The age range was 39–71 years with distribution as follows: <45 years, 24.4%; 45–54 years; 40.9%; ≥ 55 years, 34.7%. Proterinuria (defined as 1+ or higher.) was present in 15.9% of subjects at baseline. Of the Derivation cohort, 271 (8.5%) developed *Decreased GFR* at follow up.

**Table 1:** Baseline characteristics of the Derivation cohort by GFR status at follow-up

	All	Decreased GFR (*n* = 271)	Preserved GFR (*n* = 2915)	*P* value
Age, years	51.3 ± 7.4	55.1 ± 8.2	50.9 ± 7.2	<0.001
Female sex, %	29.5 (940)	15.5 (42)	30.8 (898)	<0.001
Diabetes, %	7.8 (244)	17.7 (47)	6.9 (197)	<0.001
Systolic blood pressure, mm Hg	122.3 ± 17.2	132.4 ± 20.8	121.4 ± 16.5	<0.001
Waist circumference, cm.	86.3 ± 9.6	89.9 ± 9.1	85.9 ± 9.6	<0.001
Hypertension, %	15.9 (495)	31.6 (85)	14.4 (410)	<0.001
High density lipoprotein cholesterol, mg/dL	54.6 ± 14.7	52.2 ± 13.3	54.8 ± 14.8	0.005
Triglycerides, mg/dL	146.2 ± 99.8	162.2 ± 99.9	144.7 ± 99.7	0.006
Total cholesterol, mg/dL	237.0 ± 41.8	236.1 ± 45.2	237.1 ± 41.5	0.704
Blood sugar, mg/dL	99.5 ± 25.5	109.5 ± 37.6	98.6 ± 23.9	<0.001
Body mass index, kg/m^2^	24.3 ± 3.5	25.2 ± 3.4	24.3 ± 3.5	<0.001
BMI ≥ 30 kg/m^2^, %	7.1 (222)	9.0 (24)	6.9 (198)	0.197
BMI ≥ 25 kg/m^2^, %	44.5 (1401)	57.7 (154)	43.3 (1247)	<0.001
Current smoking, %	16.2 (509)	17.5 (47)	16.1 (462)	<0.001
Cardiovascular disease, %^a^	1.3 (42)	2.6 (7)	1.2 (35)	0.056
Serum creatinine, mg/dl	1.00 ± 0.18	1.10 ± 0.18	0.99 ± 0.18	<0.001
Estimate glomerular filtration rate, mL/min/1.73m^2^	81.8 ± 12.6	73.1 ± 12.3	82.6 ± 12.4	<0.001
Categorical eGFR, mL/min/1.73m^2^				<0.001
60–74 mL/min/1.73m^2^, %	27.3 (870)	68.3 (185)	30.8 (898)	
75–89 mL/min/1.73m^2^, %	38.7 (1233)	21.8 (59)	40.3 (1174)	
90–119 mL/min/1.73m^2^, %	34.0 (1083)	10.0 (27)	28.9 (843)	
Dipstick proteinuria, %	15.9 (187)	25.2 (33)	14.8 (154)	0.002
Serum Uric acid, mg/dL	5.6 ± 1.4	6.4 ± 1.5	5.6 ± 1.4	<0.001
Hemoglobin, mg/dL	14.4 ± 1.5	14.6 ± 1.4	14.4 ± 1.5	0.046

Decreased GFR (eGFR < 60), Preserved (eGFR ≥ 60) at follow-up in 2012–2013

Data represented as mean ± SD or percent (number)

^a^Includes peripheral vascular disease, heart muscle, heart attack, stroke

#### Performance of the risk prediction models

##### Model 1(Clinical)

**Fig. 2:**
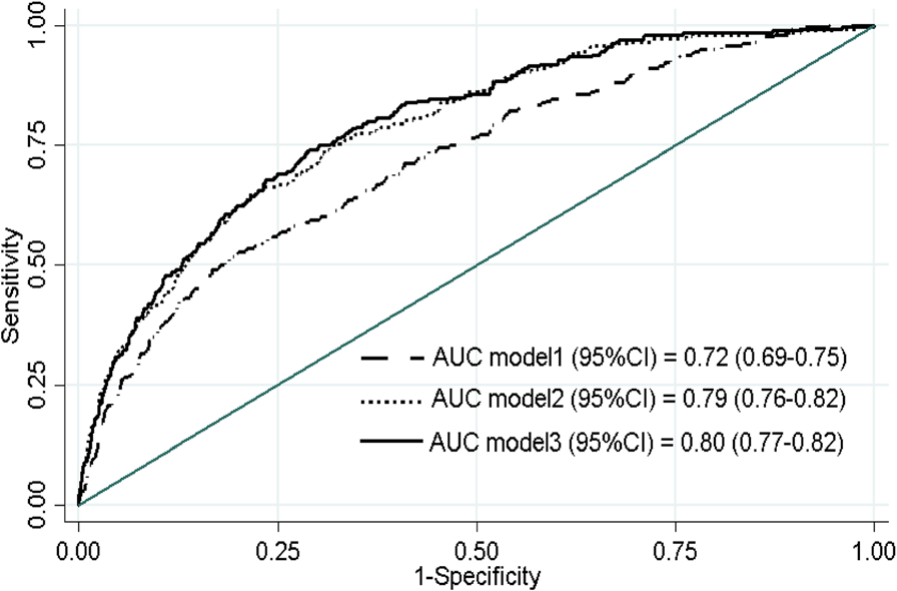
Discrimination of developing *Decreased GFR* at 10 years by different prediction models. Area under a receiver operating characteristic curve (AUC) shown for Model 1 (Clinical), Model 2 (Clinical + Limited laboratory tests), and Model 3 (Clinical + Full laboratory tests)

By univariate analysis, smoking and history of cardiovascular disease were not significant. The factors significant in the multivariate model for the simple clinical model were: Age, sex, systolic blood pressure, history of diabetes, waist circumference (Table 2) and the performance of Model 1 (Fig. 2) was good (χ^2^ = 9.02, *p* = 0.34; AUC = 0.72 (0.69–0.75), *p* < 0.001).

**Table 2:** Risk factors for developing *Decreased GFR* at 10 years

Model	Covariate	Odds ratio (95% CI)	*P*-Value
1	Age (per year)	1.06 (1.04–1.08)	<.001
	Sex (male)	1.83 (1.27–2.63)	.001
	Systolic blood pressure (per mm/Hg)	1.02 (1.02–1.03)	<.001
	Waist circumference (per cm)	1.02 (1.01–1.37)	.041
	Diabetic mellitus (Yes/No)	1.75 (1.20–2.55)	.004
2	Age (per year)	1.03 (1.01–1.05)	.002
	Sex (male)	1.44 (1.00–2.08)	.049
	Systolic blood pressure (per mm/Hg)	1.02 (1.02–1.03)	<.001
	Waist circumference (per cm)	1.01 (1.00–1.03)	.113
	Diabetic mellitus	2.50 (1.74–3.60)	<.001
	GFR (per ml/min/1.73m^2^)	0.93 (0.92–0.94)	<.001
3	Age (per year)	1.03 (1.01–1.05)	.001
	Sex (male)	1.51 (1.04–2.20)	.032
	Systolic blood pressure (per mm/hg)	1.02 (1.01–1.03)	<.001
	Waist circumference (per cm)	1.01 (0.99–1.03)	.220
	Diabetic mellitus	2.60 (1.80–3.74)	<.001
	GFR (per ml/min/1.73m^2^)	0.93 (0.92–0.95)	<.001
	Uric acid^a^	1.60 (1.12–2.25)	.009
	Hemoglobin^b^	2.04 (1.22–3.41)	.007
	HDL-cholesterol^c^	1.10 (0.77–1.57)	.607

*Decreased GFR* defined as new cases with eGFR < 60 at follow-up by the CKD-EPI formula

^a^defined as uric acid >6 for female and >7 for male

^b^defined as hemoglobin < 12 for female and <13 for male

^c^defined as HDL-cholesterol < 40 for male and <50 for female

Substitution of waist circumference with overweight as a category (BMI ≥ 25) produced similar results (Additional file 1: Table S2) with the same factors remaining predictive (χ^2^ = 8.87, *p* = 0.35; AUC = 0.72 (0.69–0.75), *p* < 0.001). Substitution of hypertension for SBP also showed similar results (χ^2^ = 10.71, *p* = 0.22; AUC = 0.71 (0.68–0.74), *p* < 0.001).

##### Model 2: (Clinical + limited laboratory tests)

In model 2, the following factors were significant: Age, Sex, SBP, DM, GFR category. Waist circumference was not significant (Table 2). Substitution of waist with BMI ≥ 25 was also not significant. Model 2 (Fig. 2) had good performance (χ^2^ = 10.87, *p* = 0.21, AUC = 0.79 (0.76–0.82), *p* < 0.001). Substitution of SBP with hypertension also produced similar results (χ^2^ = 5.62, *p* = 0.69, AUC = 0.78 (0.76–0.81), *p* < 0.001).

The AUC of Model 2 was significantly different than AUC of Model 1 (*p* = <0.001).

##### Model 3: (Clinical +full laboratory tests)

Using the full laboratory model, the following parameters were significant predictors by multivariate analysis: Age, Sex, SBP, DM, GFR, Uric acid, Hemoglobin. Waist circumference and low HDL were not significant (Table 2). Model 3 (Fig. 2) performed well (χ^2^ = 8.28, *p* = 0.41, AUC = 0.80 (0.77–0.82), *p* < 0.001). Substitution of hypertension for SBP produced similar results (χ^2^ = 5.53, *p* = 0.70, AUC = 0.79 (0.760–0.82), *p* < 0.001).

The AUC of models 2 vs model 3 were not significantly different (*p* = 0.39).

##### Addition of proteinuria to risk models

When proteinuria was added to the risk factors used in Model 1, proteinuria was a significant risk factor, but waist circumference was no longer significant if proteinuria was added (Additional file 1: Table S3). The performance of Model 1+ proteinuria was good (χ^2^ = 6.07, *p* = 0.64, AUC =0.75 (0.70–0.80), *p* < 0.001). However, although AUC of Model 1+ proteinuria tended to be better than Model 1 without proteinuria, this was not statistically significant (*p* = 0.08). Proteinuria was not a significant factor when added to model 2 or 3. (*Data not shown*).

#### Sensitivity analyses

##### Exclusion of Decreased GFR subjects with eGFR decline of less than 5 ml/min over 10 years

Among 271 subjects who developed *Decreased GFR*, 26 cases (9.6%) had eGFR decline of less than 5 ml/min/1.73m^2^ over 10 years. Exclusion of these subjects did not alter the odds ratios of the risk factors in the multivariate analysis (*data not shown*) or alter the predictive values of the Models as follows: Model 1: AUC = 0.72 (0.69–0.75), χ^2^ = 4.11, *p* = 0.847; Model 2: AUC = 0.77 (0.74–0.80), χ^2^ = 11.71, *p* = 0.165; Model 3: AUC = 0.78 (0.75–0.81), χ^2^ = 6.89, *p* = 0.549.

##### Use of four-level race variable CKD-EPI equation with Asian coefficient

When the CKD-EPI Asian formula was used to calculate eGFR, the cohort consisted of 3271 subjects with preserved GFR (eGFR ≥60) at baseline. Subsequently, 204 (6.2%) developed *Decreased GFR* at 10 years. The performance of the models using the CKD-EPI Asian equation was comparable to the two level race-variable CKD-EPI equation using coefficient for non-blacks, although there was a slight reduction in calibration for Model 2 as follows: Model 1: AUC, 0.75 (0.72–0.79), χ^2^ = 3.25, *p* = 0.918. Model 2: AUC, 0.80 (0.76–0.83), χ^2^ = 23.15, *p* = 0.003.Model 3: AUC, 0.81 (0.78–0.84); χ^2^ = 6.60; *p* = 0.580.

#### Development of Risk scores

Risk algorithms for Model 1 (waist), Model 1 (BMI) and Model 2 were converted to risk scores using dichotomized values (Tables 3 and 4). Because the performance of Model 2 and Model 3 were very similar, we did not develop a risk score for model 3 given the additional complexity.

**Table 3:** Clinical Risk scores for developing *Decreased GFR* at 10 years

	Points awarded	Risk score	10 year risk of CKD %
Model 1 (waist)			
Age (years)		-2	1
< 45	0	−1	2
45–54	2	0	2
≥ 55	4	1	3
Sex		2	4
Female	0	3	5
Male	3	4	6
Waist circumference		5	7
≤ 80 for female, ≤90 for male	0	6	9
> 80 for female,<90 for male	1	7	11
Diabetes		8	14
No	0	9	17
Yes	2	10	21
Systolic blood pressure		11	26
< 120	−2	12	33
120–129	0	13	37
130–139	1	14	49
140–149	2		
149–159	3		
≥ 160	4		
Model 1(BMI)			
Age (years)		−2	1
< 45	0	−1	2
45–54	2	0	3
≥ 55	4	1	4
Sex		2	4
Female	0	3	6
Male	2	4	7
Body mass index		5	9
< 25	0	6	11
≥ 25	1	7	14
Diabetes		8	18
No	0	9	23
Yes	2	10	30
Systolic blood pressure		11	34
< 120	−2	12	50
120–129	0		
130–139	1		
140–149	2		
149–159	2		
≥ 160	3

**Table 4:** Clinical and Limited laboratory risk Score for developing *Decreased GFR* at 10 years

	Points awarded	Risk score	year 10 risk of CKD %
Model 2			
Age (years)		−3	1
< 45	0	−2	1
45–54	2	−1	1
≥ 55	4	0	2
Sex		1	2
Female	0	2	2
Male	2	3	3
Diabetes		4	3
No	0	5	4
Yes	5	6	5
Systolic blood pressure		7	5
< 120	−3	8	7
120–129	0	9	8
130–139	1	10	10
140–149	3	11	10
149–159	4	12	13
≥ 160	5	13	14
Estimate glomerular filtration rate		14	16
≥ 90 mL/min/1.73m^2^	0	15	19
75–89 mL/min/1.73m^2^	1	≥16	≥21
60–74 mL/min/1.73m^2^	9		

The performance of the *Clinical Risk Score* (derived from Model 1) were: χ^2^ = 9.19, *p* = 0.33; AUC = 0.71 (0.68–0.74), *p* < .001, and for the *Clinical + Limited laboratory test Score* (derived from Model 2) were: χ^2^ = 11.05, *p* = 0.19, AUC =0.79 (0.76–0.82), *p* < 0.001.

The difference in discrimination (AUC) between the *Clinical* and the *Clinical + Limited laboratory test Score* was significant (*p* < 0.001).

#### Internal validation

For Model 1, the *calibration bias* was 0.00004 (*p* = 0.60). The *C* statistics of the bootstrap validation model 1 (AUC: 0.71 (0.68–0.74)) was similar to the developed Model 1.

For Model 2, the *calibration bias* was 0.06 (*p* = 0.12). The *C* statistics of the bootstrap validated model 2 (AUC: 0.75 (0.72–0.78)) was similar to the developed Model 2.

#### External validation

From a total of 2584 participants from EGAT 3 cohort recruited in 2009, we excluded participants missing serum creatinine at baseline (*n* = 9) or at follow-up in 2014 (*n* = 14), age at baseline less than 40 years (*n* = 904), participants who did not attend follow-up (*n* = 234) or died (*n* = 14) or those with eGFR < 60 at baseline (*n* = 14). The Validation cohort comprised of 1395 subjects (Fig. 1). The baseline characteristics of the participants included in the Validation cohort are shown in Additional file 1: Table S4. At 5 years, 1.9% of the Validation cohort developed *Decreased GFR*. The performance of the Risk Scores for predicting *Decreased GFR* in the Validation cohort were as follows: Model 1 (waist): AUC, 0.66 (0.55–0.78); χ^2,=^4.31, *p* = 0.229; Model 2: AUC,0.88 (0.80–0.95); χ^2,=^2.29, *p* = 0.514.

## Discussion

We developed risk prediction models for developing decreased eGFR at 10 years in a middle-age to older Thai general population using standard clinical parameters and routine laboratory tests. The predictors for the clinical model were: age, sex, systolic blood pressure, waist circumference or body mass index, and history of diabetes. The predictors for clinical and limited laboratory tests comprised of age, sex, systolic blood pressure, diabetes mellitus and baseline eGFR. The risk models demonstrated good discrimination and calibration with good internal validation. The addition of more laboratory tests of hemoglogin concentration, uric acid, HDL did not increase the performance of the clinical and limited laboratory tests significantly. Based on these results, we developed 2 simplified risk scores: a clinical risk score and a combined clinical and limited laboratory risk score. External validation using a separate cohort confirmed good performances of these scores. The parameters used in the scores are readily available for self–testing or evaluation by medical personnel in the primary care settings appropriate for a resource-limited setting such as Thailand or other parts of Asia.

Improved clinical prediction is an essential component of personalized medicine. Clinical prediction tools such as the Framingham cardiovascular risk score [6] have helped shape public health policy in the primary prevention of cardiovascular disease in many countries. However, despite the identification of several key renal risk factors, [5] similarly useful risk scores for predicting long term risk of new CKD has not been developed in an Asian general population. We are aware of 3 prior published risk prediction scores for incident CKD from community-based cohorts. The first study was derived and validated using data from middle-aged and older adults in the US community [12]. From this study, the final model included 8 variables: age, sex, anemia, hypertension, diabetes mellitus, cardiovascular disease, history of heart failure, and peripheral vascular disease. This risk score had moderate discriminatory power (c-statistic 0.70) and did not contain data on baseline GFR or proteinuria. The second study evaluated Taiwanese subjects, but was compromised by poor discriminatory power (c-statistic 0.67) and short follow-up (median 2.2 years) [28]. Because the follow-up of this study was very short, only those subjects with very rapid decline in GFR would be detected and the cumulative effects of risk factors on CKD development would be underestimated. The most recent risk score was derived from Caucasian subjects from the Framingham cohort [11]. This study shared several elements to our study including a similar follow-up period of 10 years with similar, but not identical risk predictors. In the Framingham study, age, diabetes and hypertension were significant predictors for the clinical score, and age diabetes hypertension, GFR, proteinuria were predictors in the combined clinical and laboratory model. Both the clinical and the combined clinical and laboratory tests had a high degree of accuracy and discriminatory power in US Caucasian or Black subjects (AUC 0.78–0.83). However, the Framingham risk score had low discriminatory power and accuracy when tested in our cohort (χ2 = 256.5, *p* < 0.001 and AUC 0.63 for model 2). Compared to our scoring system, age was the most significant contributor to the combined risk score in the Framingham study with diabetes, and hypertension only contributing in a minor role. In our score, both diabetes, hypertension and baseline eGFR were more important contributors to the score. In addition, being overweight was an important clinical predictor of CKD whereas this was not included in the Framingham score. The importance of obesity and diabetes highlighted in our score is especially striking given the rise in obesity and diabetes across low to middle-income populations in Asia with increasing globalization [8, 10].

Previous prediction scores for incident CKD were derived using the modification of diet in renal disease study (MDRD) equation [21]. The MDRD formula was first developed in US patients with established CKD (6). CKD-EPI equation, which was derived from both CKD and normal subject cohorts has been shown to be more accurate than MDRD especially in subjects with preserved GFR [20] Furthermore, CKD-EPI has been shown to be superior to MDRD at predicting adverse outcomes and improved the accuracy in outcome prediction in Caucasian and Black US subjects [29] There is considerable controversy on the optimum eGFR equation in Asian populations [30, 31]. There are as much as 20–30 ml/min/1.73m^2^ differences in GFR estimates between various Asian formulae. These discrepancies results in as much as 10 fold variations in CKD prevalence rate, and alter the prognostic significance attributable to the presence of CKD [32]. Differences in the reference GFR methods, and the proportion of non-CKD subjects in the development cohort likely account for these discrepancies as much as any biological differences between Asian subjects of various ethnicities [30, 31]. A Thai eGFR equation has been developed with Thai patients with established CKD [33] using a short plasma clearance of 99^Tc^ DTPA as the GFR measurement method. Given the lack of inclusion of normal subjects in the development cohort and methodological issues used to develop the Thai eGFR equation [33], we elected to use CKD-EPI for the sake of generalizability of the score to other Asian cohorts and for comparisons with other global populations [34, 35]. The rationale of our choice is supported by the fact that the CKD-EPI equation-based CKD staging has been shown to result in similar risk predictions for adverse outcomes in Asians, Whites and Blacks in a large meta-analysis [36]. In addition, we also tested the performance of our risk scores using the Asian coefficient of the four-level race variable CKD-EPI equation to calculate eGFR. Although this equation was developed in Asian populations, its role remain uncertain as the accuracy can vary in different Asian populations [26] Changing to the CKD-EPI Asian equation resulted in lower incident cases with *Deceased GFR*, but the performance of risk models were largely similar to using the original CKD-EPI equation. This suggests that the risk scores may be used to predict *Decreased GFR* when the Asian coefficient for CKD-EPI is used to calculate eGFR but the performance might be slightly reduced.

There are several potential implications of this work. First, by allowing physicians to determine an individual’s estimated risk for *Decreased GFR*, the score may inform clinical decision-making, for example to modify treatment, frequency of follow-up or institute renal primary prevention measures in high risk patients. Secondly, the use of the score may raise the profile of kidney disease among the general population, a key goal as the current CKD awareness rates is only 1.9% in Thailand [4]. Thirdly, it is noteworthy that the discrimination of the clinical risk score is already fairly high and this score could be used for focused renal screening, identifying individuals in whom creatinine measurement would be most cost-effective. Of note, although proteinuria tended to improve the discrimination of the clinical model, the improvement was slight and not statistically significant. Given the increased cost, our study suggests that routine population-based dipstick testing proteinuria may not be worthwhile. Of course, the risk score should not be used as a substitute for established urinalysis-screening intervals in people with diabetes or have other high risks. Finally, our score may be useful in estimating the individual risk and future prevalence of CKD in middle age to older subjects from other Asian general population [8, 10]. The risk factors used in our score such as older age, diabetes and hypertension are universal risk factors for CKD [10]. Many low to middle-income Asian countries are exposed to similar health impact of globalization as Thailand, and share similar prevalence of many CKD risk factors that may be considerably different from the West [37]. Combined with a closer genetic background, risk scores developed in one Asian population may be more accurate at predicting CKD in another Asian population. Although our scores have been validated externally using a separate cohort, the cohort used for validation consisted of younger Thai subjects from a similar employment background as the Derivation cohort. Participants also had shorter follow-up period of five years and a lower incidence of *Decreased GFR*. Further studies in diverse Asian cohorts with longer follow-up duration are necessary to confirm the usefulness of our score in predicting the long term risk of CKD in other Asian populations.

There are several strengths to this study. To our knowledge, this is the first prospective risk score to predict incident cases of *Decreased GFR* with follow-up of up to 10 years in an Asian population. We employed a community-based cohort with detailed assessment of risk factors and standardized calibrated enzymatic creatinine measurements. Several limitations also should be acknowledged. Baseline and follow-up creatinine were measured on a single occasion. According to KDIGO guidelines, the diagnosis of CKD requires two estimates of GFR separated by 3 months [14]. As such, the outcome we evaluated in this study does not fulfill the criteria of incident CKD, but rather, the outcome represented incident cases with decreased eGFR (eGFR < 60). This is a limitation our study shares with most published studies involved in developing incident CKD score including the Framingham heart study [11]. Multiple measurements in cohort studies are costly to perform especially in a resource-limited setting such as ours. By measuring the follow-up eGFR only once, we cannot exclude the fact that some subjects may have reversible acute kidney injury rather than persistent CKD. In addition, some subjects with a rather low borderline eGFR may have a follow-up eGFR slightly lower than 60 just due to random variation of serum creatinine. In practice, the EGAT subjects who attended the follow-up examination were not acutely ill and significant acute kidney injury was probably not frequent. Exclusion of subjects with less than 5 ml/min/1.73 m2 change in eGFR in our sensitivity analysis did not alter the performance of the risk score. Thus we expect that the risk factors identified in our study and our risk score should be valuable in identifying subjects at risk of developing incident CKD in the general population. Nonetheless, a single measurement of eGFR may lead to an overestimation of incident CKD. Future studies with repeated creatinine measurements that can confirm the presence of decreased eGFR after 3 months should provide a more accurate prediction of risks of developing incident CKD, although such a study would be more expensive to perform.

We used dipstick proteinuria rather than urine albumin creatinine ratio*.* It is possible that quantitative proteinuria measurement might have provided better prediction for *Decreased GFR* and improved our prediction models. The aim of this study was to devise scores to screen subjects at risk of *Decreased GFR* in a resource-limited setting and urine albumin creatinine ratio was not performed at baseline because of the higher expense. The KDIGO 2012 guidelines [14] suggested that urine dipstick might be substituted for albumin creatinine ratio when the latter is not available. In other scores e.g. Framingham, substitution of urine dipstick for urine albumin creatinine ratio did not alter the results [11].

A number of the participants who attended the visit in 2002–2003 did not attend the follow-up visit in 2012–13. It is not surprising that the health risk profile of those who attended both visits were statistically better than the total study population at baseline since death or retirement were common reasons for non-attendance. Although the differences in these risk factors were statistically significant, the actual differences were clinically quite small for most variables. Nonetheless, it is possible that the subjects who did not attend the follow-up examination were sicker and the true incidence of *Decreased GFR* might have been underestimated. Although the EGAT cohort is a community-based cohort, there may be some differences in the participant profiles from the Thai population as a whole. All participants were Thais and Thai-Chinese who represent the vast majority (over 95%) of the Thai Census population. Our study included only middle-age to older subjects and had a higher percentage of males than females compared to the total Thai population. EGAT employees come from all regions of Thailand and cover a wide-range of sociodemographic backgrounds [13]. Nonetheless, the socio-economic status of EGAT employees is probably better than some of the most severely economically disadvantaged Thais, and the study did not include the severely ill or disabled subjects excluded from employment. The prevalence of subjects with decreased eGFR and the CKD risk factors in our study are comparable to other a representative cross-sectional population surveys from Thailand [4]. Therefore, our risk score should applicable in assessing the risks of developing decreased GFR in community-based Thai subjects, although caution may be necessary in extrapolating findings to groups not represented in our study (for example younger or very old subjects or those who are institutionalized).

## Conclusions

We showed that clinical or combined clinical and laboratory risk models based on simple parameters available in the primary care had good accuracy and discrimination power to estimate the 10-year probability of developing *Decreased GFR* in a middle age to elderly non-institutionalized Thai general population. In addition, we validated these prediction models in a separate group of Thai subjects from a similar employment background. Additional studies are necessary to determine the validity of these scores in other Asian populations. The benefits of the two risk scores derived from these models in increasing self-awareness for CKD risks and for targeting individuals in the Thai or other Asian communities at high risk of CKD for specific interventions requires further studies.

## Additional file

Additional file 1: Table S1. Baseline characteristics of the participants with serum creatinine at both baseline (2002-2003) and follow-up (2012-2013) compared to all subjects with serum creatinine at the baseline visit. **Table S2.** Alternative clinical model (Model 1) with body mass index. **Table S3.** Clinical Model (Model 1) with proteinuria. **Table S4.** Baseline characteristics of EGAT 3 participants in the Validation dataset. (DOCX 20 kb)

## Abbreviations

AUC: Area under the curve
CKD-EPI: Chronic Kidney Disease–Epidemiology Collaboration
DBP: Diastolic blood pressure
DM: Diabetes mellitus
EGAT: The Electric Generating Authority of Thailand
ESRD: End-Stage renal disease
GFR: Glomerular filtration rate
HDL: High-density lipoprotein
HTN: Hypertensive
LDL: Low-density lipoprotein
MDRD: Modification of diet in renal disease study.
SBP: Systolic blood pressure
