# Supplementary material for: Risk scores to predict decreased glomerular filtration rate at 10 years in an Asian general population
Source: BMC Nephrol. 2017 Jul 17;18:240. doi: 10.1186/s12882-017-0653-z (PMC5512831; doi:10.1186/s12882-017-0653-z)
Supplement: Additional file 1: Table S1. — Baseline characteristics of the participants with serum creatinine at both baseline (2002-2003) and follow-up (2012-2013) compared to all subjects with serum creatinine at the baseline visit. Table S2. Alternative clinical model (Model 1) with body mass index. Table S3. Clinical Model (Model 1) with proteinuria. Table S4. Baseline characteristics of EGAT 3 participants in the Validation dataset. (DOCX 20 kb) [file 12882_2017_653_MOESM1_ESM.docx]

BNEPD1600711-**Additional file 1**

**Table S1: Baseline characteristics of the participants with serum creatinine at both baseline (2002-2003) and follow-up (2012-2013) compared to all subjects with serum creatinine at the baseline visit**

|  | Both baseline and follow-up visits | All Baseline visit | P value |
| --- | --- | --- | --- |
| n | 3441 | 5010 |  |
| Age, years | 51.7 ± 7.5 | 52.8 ± 7.6 | <0.001 |
| Female sex, % | 28.8 (991) | 26.0 (1305) | 0.005 |
| Diabetes, % | 8.2 (277) | 10.8 (531) | <0.001 |
| Systolic blood pressure, mm Hg | 122.9 ± 17.3 | 125.3 ± 18.4 | <0.001 |
| Waist circumference, cm. | 86.6 ± 9.6 | 87.4 ± 9.8 | <0.001 |
| Hypertension, % | 18.1 (608) | 21.5 (1052) | <0.001 |
| High density lipoprotein cholesterol, mg/dL | 54.4 ± 14.7 | 53.6 ±14.7 | 0.015 |
| Triglycerides, mg/dL | 146.8 ± 99.2 | 154.0 ± 11.3 | 0.02 |
| Total cholesterol, mg/dL | 237.3 ± 41.9 | 237.2 ± 43.4 | 0.926 |
| Blood sugar, mg/dL | 99.8 ± 25.1 | 103.8 ± 32.5 | <0.001 |
| Body mass index, kg/m2 | 24.4 ± 3.4 | 24.6 ± 3.5 | 0.089 |
| Current smoking, % | 15.9 (539) | 19.1 (945) | <0.001 |
| Serum Creatinine | 1.03 ± 0.21 | 1.05 ± 0.35 | 0.003 |
| eGFR, mL/min/1.73m2 | 79.66 ± 14.41 | 78.76 ± 15.08 | 0.006 |
| Dipstick proteinuria, % | 17.0 (229) | 20.4 (422) | 0.014 |
| Serum Uric acid, mg/dL | 5.7 ± 1.5 | 5.8 ± 1.5 | 0.007 |
| Hemoglobin, mg/dL | 14.4 ± 1.5 | 14.5 ± 1.5 | 0.211 |

**Table S2: Alternative clinical model (Model 1) with body mass index**

| **Covariate** | **Odds ratio (95%CI)** | ***P*-Value** |
| --- | --- | --- |
| Age (per year) | 1.06 (1.04-1.08) | <.001 |
| Sex (male) | 1.97 (1.38-2.81) | <.001 |
| Systolic blood pressure  (per mm Hg) | 1.02 (1.02-1.03) | <.001 |
| Body mass index≥25 | 1.34 (1.02-1.75) | .035 |
| Diabetic mellitus | 1.79 (1.23-2.61) | .002 |

**Table S3:** **Clinical Model (Model 1) with proteinuria**

| **Covariate** | **Odds ratio (95%CI)** | ***P*-Value** |
| --- | --- | --- |
| Age (per year) | 1.14 (1.09-1.09) | <.001 |
| Sex (male) | 1.21 (0.74-1.96) | .454 |
| Systolic blood pressure  (per mm Hg) | 1.03 (1.02-1.04) | <.001 |
| Waist circumference  (per cm) | 1.01 (0.99-1.04) | .275 |
| Diabetic mellitus | 1.45 (0.85-2.47) | .174 |
| Proteinuria | 1.65 (1.04-2.62) | .033 |

**Table S4 Baseline characteristics of EGAT 3 participants in the Validation dataset**

|  | **Validation**  **dataset** |
| --- | --- |
| n | 1395 |
| Incident CKD, % | 1.9 (27) |
| Follow up, years | 5 |
| Age, years | 45.6 ± 4.2 |
| Female sex, % | 24.5 (342) |
| Diabetes, % | 7.8 (109) |
| Systolic blood pressure, mm Hg | 121.5 ± 14.9 |
| Waist circumference, cm. | 87.3 ± 9.9 |
| Hypertension, % | 29.5 (412) |
| High density lipoprotein cholesterol, mg/dL | 51.1 ± 12.2 |
| Triglycerides, mg/dL | 138.0 ± 98.4 |
| Total cholesterol, mg/dL | 219.0 ± 39.1 |
| Blood sugar, mg/dL | 96.1 ± 26.0 |
| Body mass index, kg/m^2^ | 24.2 ± 3.6 |
| BMI≥25 kg/m^2^, % | 36.3 (501) |
| Current smoking, % | 18.2 (251) |
| Serum Creatinine | 0.9 ± 0.2 |
| Estimate glomerular filtration rate, mL/min/1.73m^2^ | 93.5 ± 12.6 |
| Categorical eGFR, mL/min/1.73m^2^ |  |
| 60-74 mL/min/1.73m^2^, % | 9.0 (125) |
| 75-89 mL/min/1.73m^2^, % | 30.4 (424) |
| 90-119 mL/min/1.73m^2^, % | 60.6 (846) |
| Dipstick proteinuria, % | 4.5 (62) |
| Serum Uric acid, mg/dL | 5.6 (1.5) |
